# The Association of *Helicobacter pylori* Biofilm with Enterovirus 71 Prolongs Viral Viability and Survival

**DOI:** 10.3390/ijms241914500

**Published:** 2023-09-24

**Authors:** Ammar M. Hassanbhai, Meng Chee Phoon, Vincent T. Chow, Bow Ho

**Affiliations:** 1 Department of Microbiology & Immunology, Yong Loo Lin School of Medicine, National University of Singapore, Singapore 117545, Singapore; hiammar@gmail.com (A.M.H.); micpmc8@gmail.com (M.C.P.); michob@gmail.com (B.H.); 2 Host and Pathogen Interactivity Laboratory, NUHS Infectious Diseases Translational Research Program, Yong Loo Lin School of Medicine, National University of Singapore, Singapore 117545, Singapore; 3 Department of Food Science & Technology, Faculty of Science, National University of Singapore, Singapore 117542, Singapore

**Keywords:** enterovirus 71, *Helicobacter pylori*, biofilms, virus viability, microbe–microbe interactions

## Abstract

The transition time during which a virus leaves its host and infects the next susceptible host is critical for virus survival. Enterovirus 71 (EV71) is stable in aqueous environments, but its molecular interactions with bacteria and their biofilms are not well-established. *Helicobacter pylori* is a highly successful gut bacterial pathogen, with its capacity to form biofilms being linked to its transmission. Given that both are gut-associated microbes, we hypothesized that biofilms formed by *H. pylori* may play a significant role in the survival of EV71 in the external environment. In this study, we examine the interactions of EV71 with the preformed biofilm of *H. pylori* to mimic its natural state in the environment. Immunofluorescence confocal microscopy and scanning electron microscopy revealed that EV71 particles persisted for up to 10 days when incubated with the *H. pylori* biofilm. Furthermore, the presence of the *H. pylori* biofilm significantly augmented viral viability, as verified through virus plaque assays. Interestingly, the viability of EV71 was dependent on the quantity of *H. pylori* biofilm formation. Thus, two *H. pylori* strains able to generate large amounts of biofilm could facilitate EV71 viability for up to 17 days, whereas two other *H. pylori* strains that produced moderate or low quantities of biofilm could not prolong virus viability. It is interesting that biofilm contains N-acetyl-glucosamine and glycosaminoglycan, and that EV71 has binding affinity to cell-surface heparan sulfate glycosaminoglycan, which acts as an EV71 attachment receptor. The synergistic ability of *H. pylori* biofilm to promote EV71 viability for extended periods implies that *H. pylori* biofilm may serve as an additional pathway of EV71 transmission.

## 1. Introduction

It is generally accepted that biofilm plays an important role during microbial growth, in addition to its planktonic or free-living counterparts. It is well-established that most microorganisms can form biofilms [1]. Whenever free-living microbial cells encounter a surface, they switch from the planktonic lifestyle to form sessile communities. Biofilm can be defined as cells that have attached to a surface or interface; these cells are embedded in a matrix of extracellular polymeric substances (EPS). Biofilm-associated microorganisms exhibit an altered phenotype with respect to their growth and gene transcription [2]. The biofilm confers protection on the microbe against attacks by other predators, such as phages; it also promotes antibiotic resistance while maintaining the microbial microenvironment [3]. In short, the biofilm protects the microorganism from external insults. Bacteria constitute the most successful form of life on planet Earth in terms of total biomass as well as the variety and extent of habitats colonized. Hence, where there is a surface with adequate moisture and suitable nutrients, biofilm populations will arise and biofilm communities will thrive and dominate [4].

Water has been implicated as a source of infection by *Helicobacter pylori*. Children from homes using a municipal water supply in Peru were three times more likely to be infected than those whose homes had internal water sources [5]. Interestingly, *H. pylori* DNA was detected in water distribution sources all over the world, including Japan, Mexico, Peru, England, Scotland, and Gambia [6,7,8,9,10,11,12,13].

The biofilm of *H. pylori* is a unique structure comprising live bacteria (both spiral and coccoid forms), dead bacteria, and EPS primarily consisting of mannose-related proteoglycans (proteomannans) [14]. The *H. pylori* biofilm is a micro-niche that protects the bacteria from external insults, prolongs their survivability, and plays a significant role in transmission to the human host. *H. pylori* biofilm can be found in relation to water sources. The survival of *H. pylori* within biofilm in the external environment may thus facilitate its cohabitation with other microbial communities. *H. pylori* can form a biofilm in association with vegetables to enhance its bacterial survivability for extended periods of time in an extra-gastric environment [15].

Viruses are not free-living organisms but remain dormant between hosts and require living cells for viral replication. Human pathogenic viruses contribute significantly to the waterborne disease burden including gastroenteritis, but they may also cause more serious diseases such as meningitis and hepatitis [16]. In particular, pathogenic enteric viruses are excreted for long periods of time and at high concentrations by infected persons, reaching up to 10^10^ infectious particles per gram of feces [16,17].

In addition to protecting its bacteria, the *H. pylori* biofilm may also support the viability of other microbes present in the environment [15]. Therefore, to validate this hypothesis, this study investigated *H. pylori* biofilm interactions with a prevalent and important organism related to the gastrointestinal route of transmission, i.e., enterovirus 71 (EV71). EV71 belongs to the human enterovirus A species of the *Enterovirus* genus within the family *Picornaviridae*. EV71 is a non-polio enterovirus and is a known cause of severe poliomyelitis-like illness, often resulting in large outbreaks worldwide [18,19,20,21]. EV71 transmission is via the fecal–oral route [22]. EV71 infection manifests most frequently as a childhood disease known as hand, foot, and mouth disease (HFMD). Transmission occurs through direct contact with saliva, feces, vesicular fluid, or the respiratory droplets of an infected person and indirectly through contaminated fomites. Before infecting their hosts, the transit time of viruses in the environment may be brief or prolonged [17]. Environmental conditions play a significant role in viral viability in the case of viruses with long transit times. Before infecting another host, virus survival and viral load are determined through the association of the virus with environmental factors. Interestingly, enteroviruses are stable for long periods in extended-aeration sludges and in oxidation-rich ditch sludges [23]. This suggests that under suitable conditions, enteroviruses are able to survive outside the host for prolonged periods. However, studies on EV71 to date have mostly focused on its epidemiology and pathologic complications, and less on its survival outside the human host.

The common theme shared between *H. pylori* and EV71 is their affinity for water. This study was performed to exemplify the role of these two pathogenic organisms as a model of microbe–microbe interactions. *H. pylori* is estimated to infect about half of the world’s population, rendering it one of the most common microbial pathogens [24]. The detection of *H. pylori* using PCR in water distribution systems and water pots indicates water as a potential source of *H. pylori* [8,11,12,13]. More importantly, *H. pylori* is capable of forming biofilms in the context of in vitro cultures [14,25,26,27,28].

If the interaction between viruses and biofilms is limited to attachment, it may imply that biofilms aid in the adsorption of viruses and thus restrict their spread. However, the biofilm life cycle starts with attachment, while the final stage involves the detachment of the biofilm to colonize new areas. Together with this sloughed biofilm are the attached viruses. The quantity of any contaminating viruses would decline during prolonged external environmental conditions, which may be exacerbated through treatment with chemicals, heat, or irradiation [17]. However, when associated with the biofilm, the virus may be shielded from hostile environments until it infects its susceptible host. We propose that biofilms may offer a means of protection to the virus from harsh environments, which can augment virus survivability. Given that both biofilms and enteric viruses are ubiquitous, we hypothesize that certain viruses associate synergistically with bacterial biofilms to facilitate viral transmission and survivability in the external environment before the virus reaches its vulnerable host. In particular, this study focused on investigating the cooperation between EV71 and the preformed biofilms of *H. pylori* to mimic their natural state in the external environment. Preformed biofilm refers to biofilm that was formed beforehand (e.g., dehydrated biofilm).

## 2. Results

### 2.1. EV71 Particles Associate with the Preformed Biofilm of H. pylori

Following the liquid culture of *H. pylori* in the wells, crystal violet staining was performed to detect and quantify biofilm formation (as described in Section 4.2). At the air–liquid interface of each well, the stained ring represents bacteria that have adhered to the well surface. Similarly stained by crystal violet are the adherent bacteria at the bottom of each well. Together, they constitute the bacterial biofilm, which is stained by crystal violet. Crystal violet staining is a well-established method for the detection and quantification of *H. pylori* biofilm. The supernatant in each well represents the unattached, free-living planktonic bacteria [14,28].

**Figure 1:**
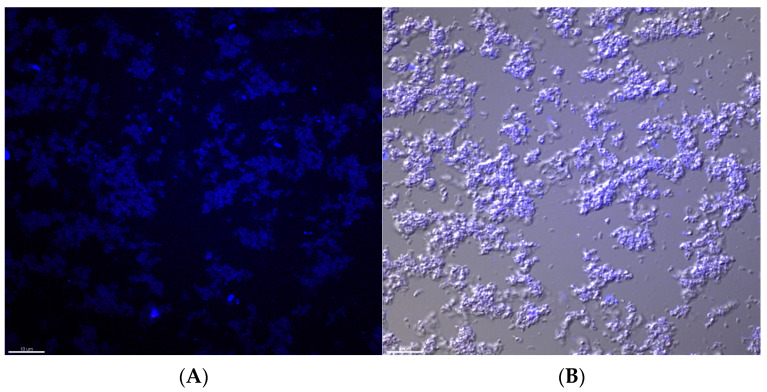
Characterization of the biofilm of the *H. pylori* NCTC11637 bacterial strain. Crystal violet staining was used to detect and quantify the biofilm produced by *H. pylori* cultured in liquid BHI-CD medium. (**A**) Calcofluor staining to detect the bacterial extracellular polysaccharides (EPS) of one-week-old *H. pylori* biofilm. (**B**) Superimposed images of bright-field microscopy and Calcofluor staining to display the location of EPS in relation to the *H. pylori* bacteria and biofilm. Scale bar represents 10 µm.

Figure 1A shows one-week-old *H. pylori* biofilm stained with Calcofluor, which is a commonly used biofilm stain that detects bacterial polysaccharides in the EPS. The same image was taken under bright-field microscopy and superimposed onto the immunofluorescence image to demonstrate the specificity of the Calcofluor stain to the *H. pylori* biofilm (Figure 1B).

**Figure 2:**
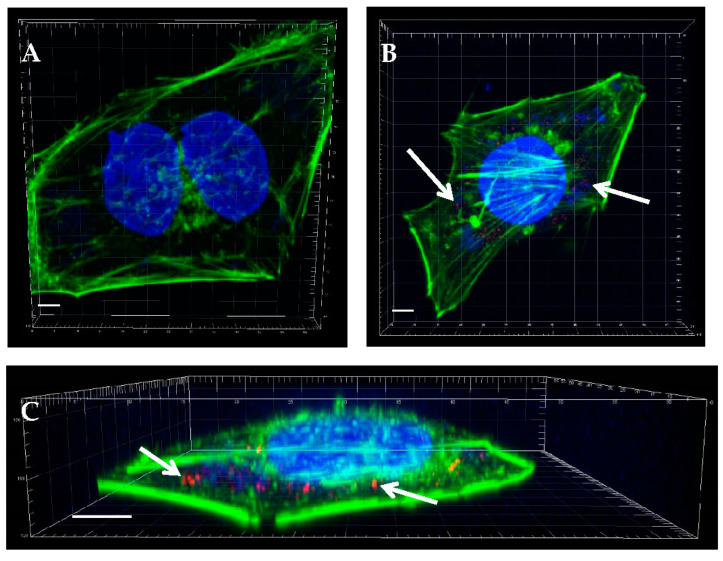
Specific immunofluorescence staining of the EV71 infection of RD cells. (**A**) Uninfected control RD cells. (**B**) RD cells were infected with EV71 at a multiplicity of infection of 1, and imaged using confocal microscopy at 6 h post-infection. (**C**) Cross-sectional three-dimensional view of one representative infected cell depicting the staining of DNA (blue by DAPI) and actin (green using Alexa Fluor 488 phalloidin). EV71 particles appear red from immunolabeling with an EV71-specific primary antibody followed by a secondary antibody conjugated to Cy3. Arrows point to exemplify immunolabeled red virus particles, which are more prominent in the three-dimensional image in (**C**) compared with the two-dimensional image in (**B**). Scale bar represents 5 µm. Images were rendered using Imaris.

Immunofluorescence staining with a specific primary antibody against the VP1 capsid protein of EV71, followed by a secondary antibody conjugated to Cy3, was employed to detect red-stained EV71 particles (Figure 2). Following the EV71 infection of RD cells for 6 h, virus particles stained red were observed within the infected cells (Figure 2B,C). However, the red-stained virions were absent from the uninfected control RD cells (Figure 2A), thus verifying the specificity of the EV71 antibody.

**Figure 3:**
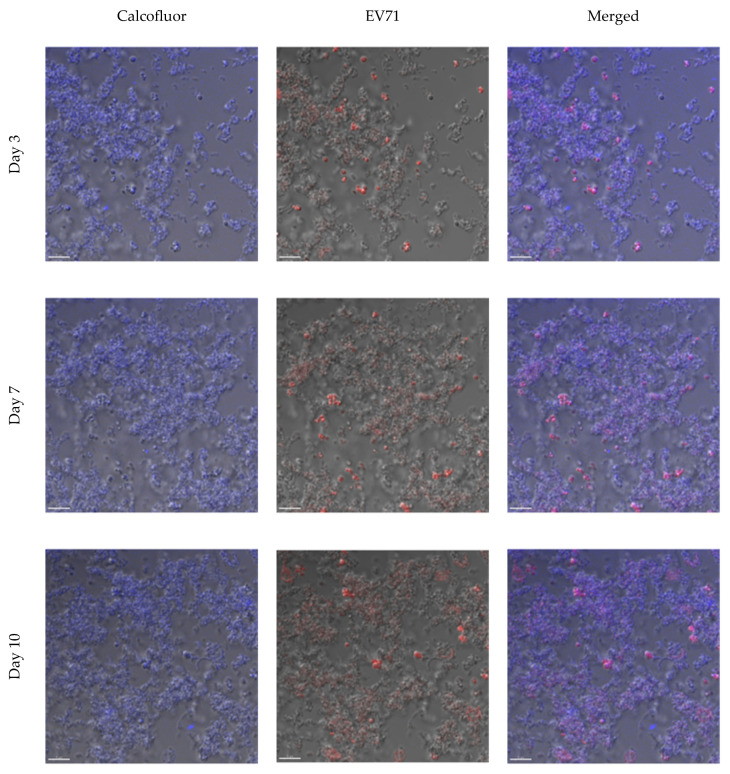
Immunofluorescence staining of the one-week-old biofilm of *H. pylori* NCTC11637 co-incubated with EV71 over durations of 3, 7, and 10 days. Calcofluor stains the biofilm EPS blue (in the left panels). EV71 particles appear red from immunolabeling with an EV71-specific primary antibody followed by a secondary antibody conjugated to Cy3 (in the center panels). The corresponding bright-field microscopic images in the left and center panels are merged to illustrate the co-localization of EV71 with *H. pylori* biofilm EPS (in the right panels). The scale bar represents 10 µm (bottom left of the panels).

To further confirm the ability of EV71 to associate with the *H. pylori* biofilm, the immunofluorescence staining of a one-week-old biofilm co-incubated with EV71 was examined. When EV71 was co-incubated with the one-week-old biofilm for 3 days, the viruses (red immunolabeled signals) were observed to associate with the biofilm (stained blue), and EV71 was still detectable after co-incubation for 7 and 10 days (Figure 3). It was noteworthy that virus detection (represented by red immunolabeled signals) did not decrease over the 10-day co-incubation period (Figure 3, bottom three panels). Furthermore, from the cross-section of the biofilm, there was no observable preference for the location (e.g., the top or bottom of the biofilm) where the virus associated with the biofilm (Supplementary Figure S1), suggesting the random binding of EV71. This experiment demonstrates the presence of EV71 in the biofilm but does not determine virus viability. EV71 was detected based on immunofluorescence, using an antibody specific to the EV71 VP1 coat protein. Hence, this assay does not discriminate between viable versus noninfectious viruses. Nevertheless, these data suggest the persistence of the virus for prolonged periods within the *H. pylori* biofilm.

**Figure 4:**
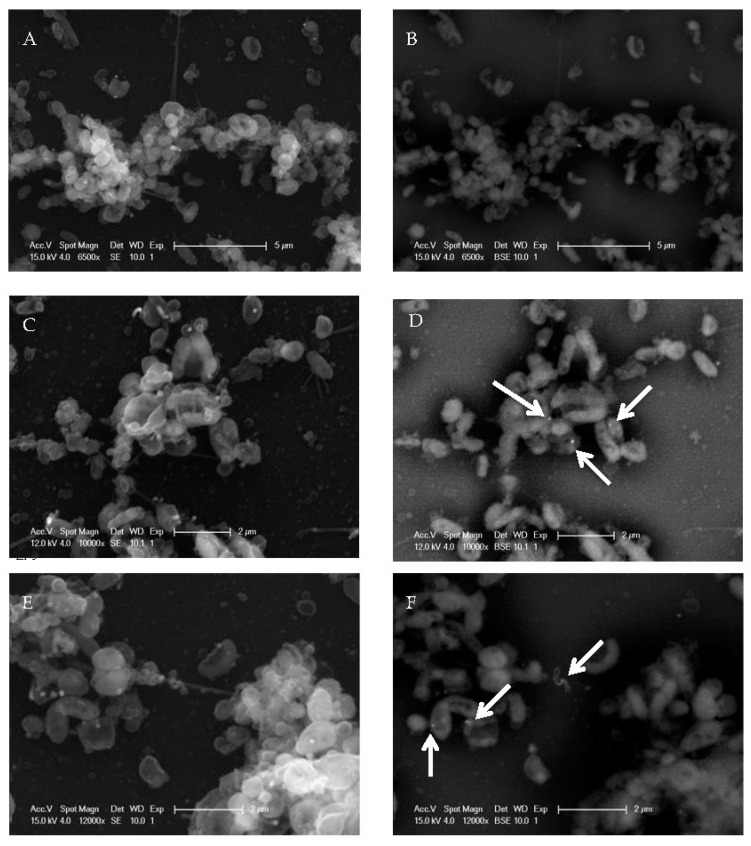

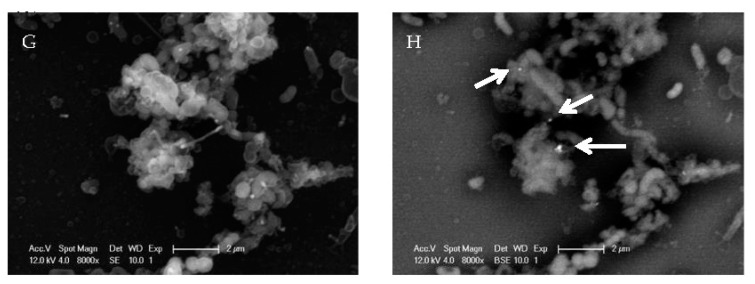
Scanning electron micrographs of the co-incubation of the preformed biofilm of *H. pylori* with EV71. EV71 was co-incubated with the one-week-old biofilm of *H. pylori* NCTC11637 for varying durations and probed for EV71 using 20 nm gold particles (as described in Section 4.5). Micrographs (**A**,**C**,**E**,**G**) were obtained using the standard electron (SE) mode. Images (**B**,**D**,**F**,**H**) were acquired using the backscatter electron (BSE) mode in order to visualize the 20 nm gold particles. Micrographs (**A**,**B**) served as the non-virus-infected control and comprised the one-week-old biofilm probed with the EV71 primary antibody and gold-conjugated, secondary antibody. Micrographs (**C**) to (**H**) represent one-week-old biofilms incubated with EV71 for varying periods: (**C**,**D**) 1 day; (**E**,**F**) 3 days; and (**G**,**H**) 1 week. This *H. pylori* strain generated prominent three-dimensional, thick, and dense biofilm structures, with multi-layered microcolonies. (**D**,**F**,**H**) Arrows indicate the electron-dense, round spots at the location of the EV71 antigen that represent EV71 virions labeled with gold particles. Representative images of each sample (with scale bars) are depicted.

To further verify the association of the EV71 particles with the biofilm formed by the *H. pylori* strain NCTC11637 at a high resolution, immunogold labeling for EV71 detection was carried out, along with scanning electron microscopy (SEM). The standard electron (SE) mode was utilized to visualize the sample, while the backscatter electron (BSE) mode was employed to detect electron-dense gold particles in labeled samples. In Figure 4, the left panels depict the standard SEM images, while the right panels portray the corresponding sample frames taken in the BSE mode. The non-virus-infected control sample under the BSE mode did not show any electron-dense gold particles in the background, indicating the specificity of immunogold labeling (Figure 4A,B). This *H. pylori* strain generated prominent three-dimensional biofilm structures that are thick and dense, with multi-layered microcolonies. This biofilm also exhibited compact attachment and adhered well to the substratum. To observe the association of EV71 with the biofilm, the one-week-old biofilm was co-incubated with EV71 for varying durations, and EV71 was detected using an immunogold-labeled, virus-specific antibody. After 1 day of co-incubation of EV71 with the *H. pylori* biofilm, virus particles were observed as electron-dense, round spots at the location of the EV71 antigen (arrows in Figure 4D). Virus particles were also detected as electron-dense round spots after EV71 co-incubation with the biofilm for 3 days and 1 week (arrows in Figure 4F,H, respectively). These electron micrographs (acquired using the BSE mode) reveal that the EV71 particles associated with *H. pylori* biofilm and were detectable even after 1 week of co-incubation.

### 2.2. The Duration of Virus Viability Is Dependent on the Quantity of Biofilm Formation

**Figure 5:**
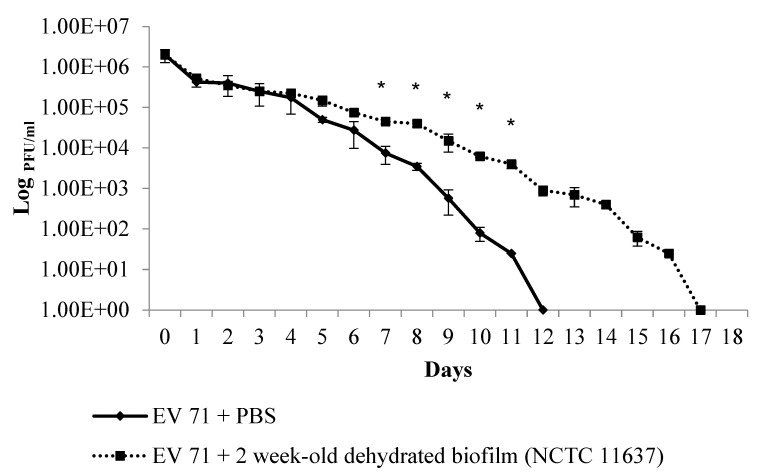
Quantification of infectious EV71 particles over a prolonged period in PBS control alone versus PBS with the preformed biofilm of the *H. pylori* strain NCTC11637. EV71 (2 × 10^6^ plaque-forming units or PFU) was added to PBS only, without biofilm (continuous line graph) or PBS with two-week-old, dehydrated *H. pylori* biofilm (dotted line graph), and virus viability was quantified daily using the virus plaque assay. *H. pylori* biofilm could extend EV71 viability for a significantly longer period of time (17 days), i.e., an additional 5 days more than EV71 incubated with PBS alone without biofilm (12 days). Viable virus titer is represented as log PFU per milliliter (PFU/mL), e.g., 1.00+E03 = 10^3^. Error bars depict standard errors derived from three independent experiments. Statistical analyses were conducted using the two-tailed Student’s *t*-test. Asterisks (*) denote *p*-values < 0.05, which were considered statistically significant (for differences from day 7 to day 11).

Next, to examine virus viability in the *H. pylori* biofilm, EV71 was incubated in PBS control alone (without biofilm) and in PBS along with the two-week-old biofilm of the *H. pylori* strain NCTC11637. In the latter sample, the supernatant, together with the biofilm, in the well was harvested and subjected to daily virus plaque assays for 18 days. Similarly, plaque assays were also performed on the control sample (EV71 in PBS alone). It was found that EV71 viability in PBS alone gradually decreased over time outside human host cells (RD cells), with infectious virus no longer being detected at 12 days (Figure 5). However, in PBS in the presence of the *H. pylori* biofilm, the infectious virus could still be recovered for a significantly longer period, i.e., up to 17 days (Figure 5). These findings indicate that the *H. pylori* biofilm is able to extend EV71 viability for a significantly longer period of time, i.e., an additional 5 days more than EV71 incubated with PBS alone (without a biofilm).

**Figure 6:**
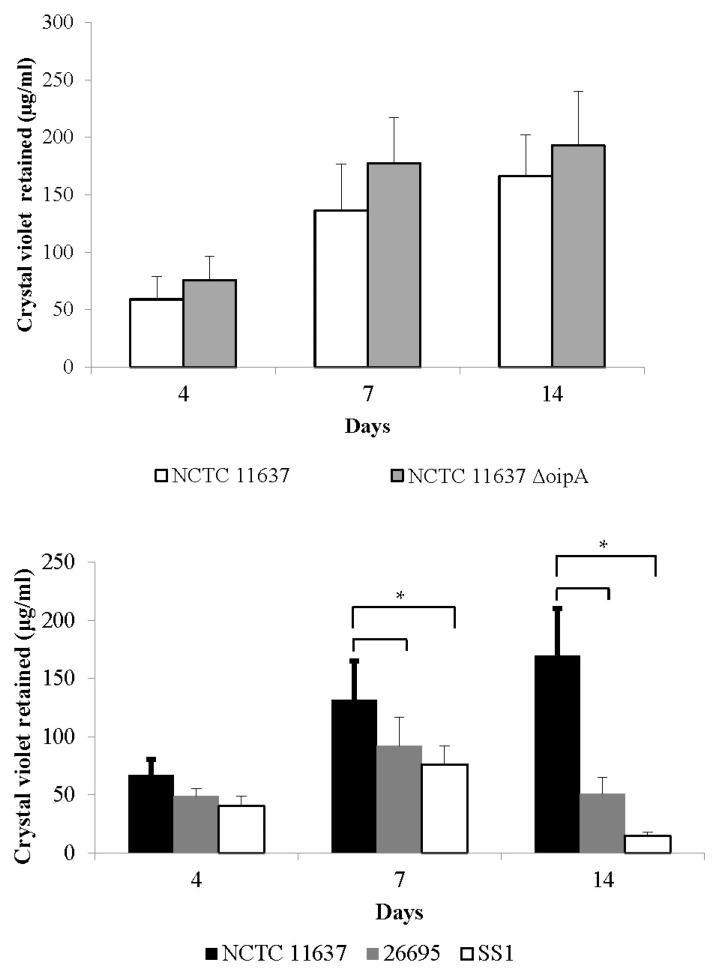
Comparison of biofilm formation between different *H. pylori* strains over a period of 14 days. The formation of the biofilm at 4, 7, and 14 days was quantified using the crystal violet assay. Biofilm production was quantified as the concentration of crystal violet retained in the biofilm (expressed in µg/mL). The highest quantities of biofilm were generated by the NCTC11637Δ*oip*A isogenic mutant and NCTC11637 strains (upper panel). Strains 26695 and SS1 were moderate and poor biofilm producers, respectively, especially at 14 days, i.e., where we noted about 3-fold and 10-fold less biofilm than that observed for NCTC11637 (lower panel). The data represent mean values and standard errors derived from duplicates of three independent experiments. Statistical analyses were conducted using Student’s *t*-test. Asterisks (*) denote *p*-values < 0.05, which were considered statistically significant.

To examine the effects of the bacterial biofilm on EV71 viability, co-incubation experiments of EV71 with biofilms generated by four different *H. pylori* strains were conducted. These strains were selected to represent a range of biofilm producers (in descending order of biofilm-forming ability: the NCTC11637Δ*oip*A isogenic mutant, NCTC11637 26695, and SS1) based on the crystal violet staining assay (Figure 6). For example, after a 14-day period, the biofilms generated by NCTC11637Δ*oip*A and NCTC11637 were at least 3-fold and 10-fold greater than those produced by the strains 26695 and SS1, respectively (Figure 6, upper and lower panels). The highest biofilm producer was NCTC11637Δ*oip*A (Figure 6, upper panel), a *H. pylori* isogenic mutant with the deletion of the *oip*A gene, which encodes the outer inflammatory protein A [29]. In this batch of experiments, the culture supernatant and the biofilm in each well were harvested separately and subjected to virus plaque assays. The objective was to compare the viability of EV71 when co-incubated with a culture supernatant (representing unattached, free-living, planktonic bacteria) versus the viability of EV71 when co-incubated with the *H. pylori* biofilm.

**Figure 7:**
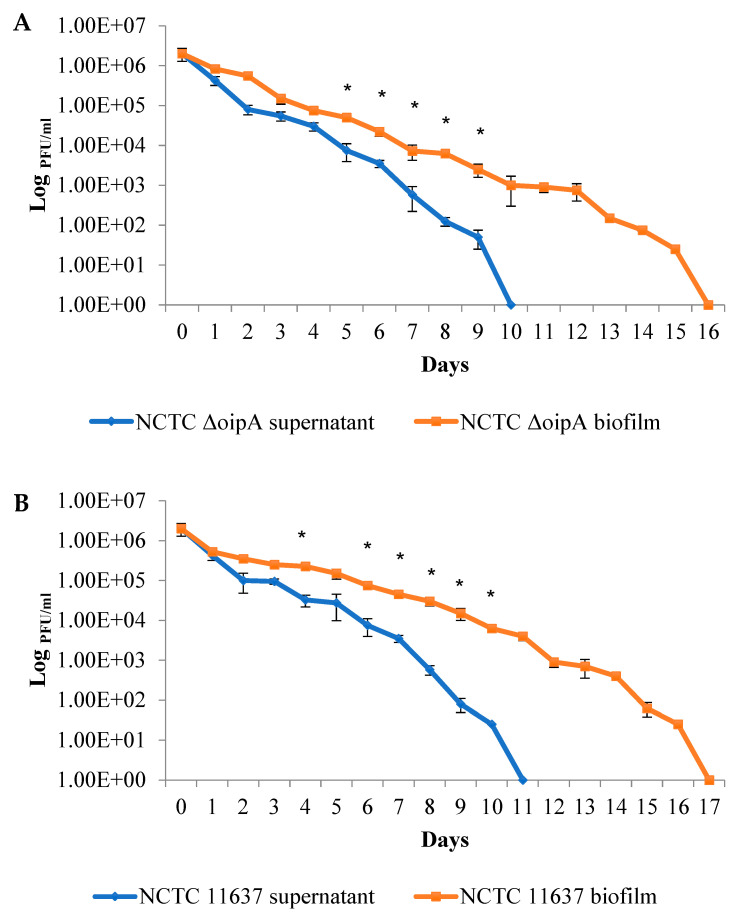

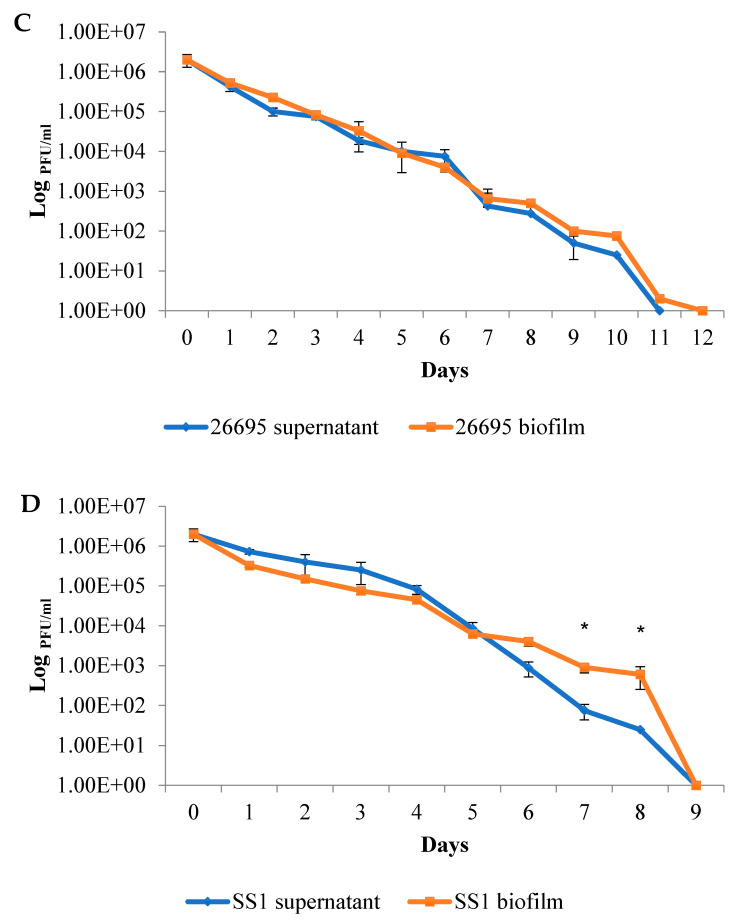
Survival of infectious EV71 in association with biofilms generated by different *H. pylori* strains. The virus plaque assay was employed to quantify infectious virus particles in association with the unattached, free-living, planktonic bacteria (culture supernatant) and with two-week-old, dehydrated biofilms of *H. pylori* strains: (**A**) NCTC11637Δ*oip*A isogenic mutant, (**B**) NCTC11637, (**C**) 26695, (**D**) SS1. The supernatant and the biofilm in each well were harvested separately and subjected to virus plaque assays. The viable virus titer is represented as log PFU per milliliter (PFU/mL), e.g., 1.00+E03 = 10^3^. The blue graphs depict the viable virus titers over time for supernatant samples, while the red graphs depict viable virus titers for biofilm samples. Error bars depict standard errors derived from three independent experiments. Asterisks (*) denote *p*-values < 0.05, which were considered statistically significant. Infectious virus could be recovered from relatively abundant biofilms produced by NCTC11637Δ*oip*A isogenic mutant and NCTC11637 strains up to 16 and 17 days, respectively, compared with the viable virus isolated from their corresponding supernatants up to only 10 and 11 days. There was little or no difference in the duration of viral viability in the biofilm and supernatant samples of the *H. pylori* strains 26695 and SS1.

In the presence of large amounts of preformed biofilm samples derived from the NCTC11637Δ*oip*A isogenic mutant and NCTC11637, infectious EV71 was recovered at up to 16 and 17 days, respectively. In contrast, the EV71 viability in the supernatant samples of NCTC11637Δ*oip*A and NCTC11637 was significantly shorter, i.e., only 10 and 11 days, respectively (Figure 7A,B). Interestingly, however, in the virus co-incubation experiments with the biofilms of strain 26695 (moderate biofilm producer) and strain SS1 (poor biofilm producer), there was little or no significant difference in the duration of EV71 viability between the biofilm samples and supernatant samples of these two strains (Figure 7C,D). Taken together, these results indicate that high quantities of *H. pylori* biofilm are able to significantly prolong and sustain EV71 viability.

## 3. Discussion

In this study, the clinically important EV71 was investigated, since it is ubiquitous in nature and constitutes a source of persistent infections worldwide [18,19,20]. EV71 interactions with dehydrated *H. pylori* biofilms were analyzed to mimic their natural state in external environments outside the human host. Our initial confocal microscopy data revealed that EV71 was able to associate with the *H. pylori* biofilm. Electron microscopy further confirmed this observation with EV71 particles adhering to the *H. pylori* biofilm surface, thus reinforcing similar reports of the attachment of other viruses (such as poliovirus and bacteriophages) to the biofilms of water distribution systems [30,31,32]. A possible explanation is that the N-acetyl-glucosamine and glycosaminoglycans content of *H. pylori* biofilms [25] may facilitate the attachment and subsequent infectivity of EV71. Intriguingly, EV71 binds to cell-surface heparan sulfate glycosaminoglycan, which serves as an EV71 attachment receptor [33]. Moreover, EV71 exhibits a high adsorption capacity to nickel-chitosan beads and retains its viral antigenicity and infectivity after desorption. Chitosan is a linear polysaccharide composed of N-acetyl-D-glucosamine and deacetylated D-glucosamine [34].

Interestingly, viable EV71 was recovered for up to 12 days when suspended in PBS alone, indicating that this virus is stable and can survive for a prolonged period outside the human host. Enteric viruses, such as norovirus and astrovirus, are also highly stable and can survive water treatment procedures and persist, thus posing risks to the users of recreational waters [35]. However, EV71 remained viable for up to 17 days when co-incubated with a two-week-old biofilm, thus providing evidence that the *H. pylori* biofilm was able to preserve and/or protect EV71 particles from degradation or inactivation. In contrast to previous studies, which employed molecular methods to determine viral levels, our findings are significant because EV71 viability was verified using virus plaque assays.

In addition, we compared EV71’s association or adhesion to the biofilms derived from four *H. pylori* strains with varying biofilm-forming abilities. We hypothesized that if the biofilm was indeed responsible for preserving viral activity, then more biofilm would logically provide greater protection and thus enhance virus viability, and vice versa. In the presence of large amounts of *H. pylori* biofilm from the NCTC11637Δ*oip*A isogenic mutant and NCTC11637 strains, higher levels of infectious EV71 were recovered, implying that more biofilm sites are available for EV71 to adhere. In contrast, in *H. pylori* strains 26695 and SS1 with relatively lower biofilm-forming ability, the availability of adherence sites would be diminished. Under the latter conditions, EV71 would remain unbound in the supernatant, culminating in a more rapid decline in its viability. Our findings strengthen the notion that certain bacterial biofilms indeed confer considerable protection to specific viruses, rendering the latter viable and stable for prolonged periods outside their human host. *H. pylori* can survive in water systems by associating with autochthonous organisms present in biofilms in such systems [36]. Enterovirus has also been detected in water distribution systems [37,38,39]. Given that both *H. pylori* and EV71 exist in water distribution systems [8,10,11], it is important to highlight the innate ability of *H. pylori* to generate biofilms that can associate with EV71 to promote viral transmission and survival. Furthermore, the interactions of EV71 VP1, VP2, VP3, and VP4 proteins to self-assemble the icosahedral capsid also enable this non-enveloped virus to tolerate the environment of the gastrointestinal tract [40,41]. Our findings shed some light on how bacterial biofilms in drinking water distribution systems (e.g., reservoirs, pipes, streams, lakes) may interact with resident viruses, with important implications for public health.

Viruses represent a significant cause of recreationally associated waterborne diseases [42,43]. Epidemiological studies demonstrate significant gastrointestinal, respiratory, ear, and skin infections among those who engage in water-based recreational activities [44,45,46]. Studies reveal the presence of human-enteric viruses in recreational waters and/or show positive correlation between swimming in recreational waters and a heightened risk of disease [47,48,49,50]. Although waterborne epidemics are well-documented worldwide, virus outbreaks attributed to the consumption of drinking water may be greatly underestimated [51].

The stability of an enteric virus is of fundamental significance to its transmission. First, virus stability may affect the period during which virus contamination remains a threat in the environment (e.g., in sea or fresh water or when drying on a surface). Second, it influences the efficiency of attempts to deliberately destroy such viruses, e.g., during food or water processing (sensitivity to temperature and chlorination) [17].

Pathogenic enteric viruses are excreted in large quantities by infected persons, and these viruses are discharged in wastewater [52]. In particular, enterovirus and norovirus have been demonstrated in natural wastewater biofilm [53]. Infectious viruses persist longer when associated with solids rather than being free in the water [54,55,56]. These findings suggest the possibility of the biofilm protecting the virus and its genome from degradation as well as preserving viral particles from inactivation [51,57]. Viruses may be released into external environments as single virus particles or in association with biofilms [53]. Viruses may also be released with changing physicochemical conditions of the water (e.g., temperature, pH, ultra-violet light), so that the fate of viruses may depend on their molecular interactions with biofilms [53]. In addition to EV71, many enteric viruses (such as other enteroviruses, rotavirus, norovirus, astrovirus, and hepatitis A and E viruses) are non-enveloped and contain RNA genomes, and their association with microbial biofilms may also confer protection to their virions. In future studies, it would be interesting to investigate the interactions of *H. pylori* biofilm with these non-enveloped enteric viruses. Taken together, our findings support the potential role of bacterial biofilms in enhancing virus persistence in external environments.

## 4. Materials and Methods

### 4.1. Bacterial Strains and Culture Conditions

The *H. pylori* strains NCTC11637 (ATCC 43504), 26695 (ATCC 700392), and SS1 (Sydney Strain 1) were used in this study. We also included an isogenic mutant strain NCTC11637Δ*oip*A, which was constructed previously [29]. The bacteria were cultured on chocolate blood agar for 3 days before use. Bacteria were also cultured in BHI-CD liquid media consisting of a brain heart infusion (Oxoid, Basingstoke, UK) supplemented with 0.4% yeast extract (Oxoid) and 1% β-cyclodextrin (Sigma-Aldrich, St Louis, MO, USA). All cultures were incubated under microaerophilic conditions at 37 °C with 10% CO_2_ and 95% humidity in a water-jacketed incubator (Forma Scientific, Waltham, MA, USA).

### 4.2. Preparation of Dehydrated Bacterial Biofilm and Quantification through Crystal Violet Staining

*H. pylori* strains grown on blood agar plates were transferred and resuspended in BHI-CD media using cotton swabs (Copan, Murrieta, CA, USA). Each bacterial suspension (2 mL) was then added to each well of a 12-well culture plate (Nunc, Roskilde, Denmark) and incubated under culture conditions as described above. After a period of biofilm growth (e.g., two weeks), each well was washed twice in PBS, and 70% ethanol was added to cover the biofilm for 4 h. The ethanol was then removed, and the biofilm was air-dried overnight and was ready for use in subsequent experiments.

At various time intervals, the quantification of biofilms formed in 12-well culture plates was performed as previously described with modifications [14,58]. Briefly, the culture medium was removed from each well and washed with PBS to remove non-adherent cells. After this, 2 mL of 0.5% crystal violet solution was added to stain the biofilm at room temperature. After 10 min, the excess crystal violet was removed, each well was then washed twice with PBS, and the biofilms were decolorized with 2 mL of 33% acetic acid. The optical density of the destained solution was read at 590 nm using a DU-640B UV spectrophotometer (Beckman Coulter, Indianapolis, IN, USA). The growth medium without bacterial inoculum at the corresponding time-point served as a negative control. Biofilm production was quantified using a correlation of absorbance at 590 nm (A_590_) to the concentration of crystal violet retained in the biofilm. The absorbance of the stained acetic acid eluate was measured and compared with a standard graph of A_590_ against a range of crystal violet concentrations.

### 4.3. EV71 Strain, Cell Line, and Virus Plaque Formation Assay

Enterovirus 71 strain 41 (5865/SIN/00009) is a clinical isolate from a patient who succumbed during the HFMD epidemic in Singapore in 2000, with its genome sequence deposited in GenBank under the accession number AF316321. Approval to use this strain for research purposes was granted under the Infectious Diseases Act, Singapore [59,60]. Rhabdomyosarcoma (RD) cells (ATCC CCL-136) are highly permissive for EV71 replication with a prominent cytopathic effect and were used in this study. Uninfected control and infected RD cells were cultured in minimum essential medium (Gibco, New York, NY, USA) supplemented with 2% HEPES (Gibco), 7.5% sodium bicarbonate (Gibco), and 10% fetal bovine serum (FBS) (Gibco). RD cell cultures were incubated at 37 °C with 5% CO_2_ and 95% humidity in a water-jacketed incubator (Forma Scientific).

One day prior to the virus plaque assay, RD cells were seeded into 24-well plates (Nunc) at a density of 5 × 10^5^ cells/mL. For each sample, ten-fold serial dilutions (10^−1^ to 10^−6^) in culture medium were performed. An aliquot of 200 μL of diluted sample was added to each well in duplicate (i.e., 12 wells were used per sample). The plates were then incubated for 1 h. During this time, Avicel was warmed to 37 °C. The supernatants were then removed before adding 1 mL of 1% Avicel in culture medium supplemented with 2% FBS into each well and incubated for another 3 days. The supernatants were then removed, and each well was washed twice with PBS to remove the excess Avicel. Then, 1 mL of 20% paraformaldehyde (Merck, Burlington, MA, USA) was added to each well to fix the cells for 1 h at room temperature. The paraformaldehyde was then removed, and 1 mL of 0.5% crystal violet (Sigma) was added to each well and stained for 20 min. After the removal of the crystal violet, the wells were washed thoroughly in water. The virus plaques were enumerated using the following formula: PFU/mL = number of plaques formed × dilution factor × (1000/200) [61].

### 4.4. Immunofluorescence Staining and Confocal Microscopy to Image the H. pylori Biofilm and EV71

The *H. pylori* biofilm or RD cells (with or without EV71 infection) were cultured on 13 mm borosilicate glass slides (a substrate for biofilm formation and adherent RD cells). The culture medium was removed, and the glass slides were washed in PBS. The samples were fixed in 4% paraformaldehyde for 45 min and permeabilized with 0.2% Triton X-100 (Bio-Rad) for 10 min. Mouse monoclonal anti-enterovirus primary antibody (M7064, Dako, Glostrup, Denmark) was added at a dilution of 1:10 in 0.2% bovine serum albumin in PBS (BSA-PBS). The goat anti-mouse IgG Cyanine3 conjugate secondary antibody (A10521, Invitrogen, Carlsbad, CA, USA) was added at a dilution of 1:100 in 0.2% BSA-PBS. The samples were incubated for 1.5 h for each antibody and were washed with PBS between each incubation. To stain the filamentous actin of the RD cells, Alexa Fluor 488 phallodin (Invitrogen) was added at a dilution of 1:100 in 0.2% PBS-BSA for 20 min. To stain the biofilm EPS, the slides were washed in PBS before adding 300 μg/mL of Calcofluor (Sigma) for 20 min, followed by washing with PBS. All incubations were performed at room temperature. Finally, the samples were mounted onto slides using VECTASHIELD Antifade medium (Vector Laboratories, Newark, CA, USA) and viewed using an Olympus FV1000 confocal laser scanning microscope [62].

### 4.5. Scanning Electron Microscopy and Gold Labeling of Virus Particles

*H. pylori* biofilm samples were prepared, and a primary fixative comprising 4% paraformaldehyde and 0.1% glutaraldehyde (Merck) was added to the sample. After 3 h of fixation, the samples were washed thrice in PBS for 5 min per wash. The residual aldehyde was inactivated by incubating the samples in 0.05 M glycine (Merck) in PBS for 15 min. Nonspecific interactions were blocked using freshly prepared 5% BSA in PBS for 30 min at room temperature. This was followed by three washes in 0.1% BSA in PBS for 10 min per wash. A mouse monoclonal anti-enterovirus primary antibody (M7064, Dako) was added at a dilution of 1:10 in 0.2% BSA in PBS and incubated overnight at 4 °C. This was followed by three washes in 0.1% BSA in PBS for 10 min per wash. A goat anti-mouse gold conjugate (20 nm gold particles) secondary antibody (Ted Pella, Redding, CA, USA) was added at a dilution of 1:50 in 0.2% BSA in PBS and incubated for 2 h at room temperature. The treated samples were then washed thrice in 0.1% BSA in PBS for 10 min per wash. This was followed by another three washes in PBS for 10 min per wash. The samples were then processed for SEM as described previously [14]. Briefly, the samples were subjected to fixation with 2% glutaraldehyde and 2% osmium tetroxide, washed and dehydrated in a graded ascending ethanol series. The samples were placed in a CPD 030 critical point dryer (Bal-Tec, Los Angeles, CA, USA) and then viewed at 10 kV using a Jeol JSM 5600LV scanning electron microscope [62].

## Data Availability

Not applicable.

## Supplementary Materials

The following supporting information can be downloaded at: https://www.mdpi.com/article/10.3390/ijms241914500/s1.

## References

[1] Sauer K., Stoodley P., Goeres D.M., Hall-Stoodley L., Burmølle M., Stewart P.S., Bjarnsholt T. (2022). The biofilm life cycle: Expanding the conceptual model of biofilm formation. Nat. Rev. Microbiol..

[2] Flemming H.C., van Hullebusch E.D., Neu T.R., Nielsen P.H., Seviour T., Stoodley P., Wingender J., Wuertz S. (2023). The biofilm matrix: Multitasking in a shared space. Nat. Rev. Microbiol..

[3] Yonezawa H., Osaki T., Kamiya S. (2015). Biofilm formation by Helicobacter pylori and its involvement for antibiotic resistance. Biomed. Res. Int..

[4] Krzyżek P., Grande R., Migdał P., Paluch E., Gościniak G. (2020). Biofilm formation as a complex result of virulence and adaptive responses of Helicobacter pylori. Pathogens.

[5] Klein P.D., Graham D.Y., Gaillour A., Opekun A.R., Smith E.O. (1991). Water source as risk factor for Helicobacter pylori infection in Peruvian children. Lancet.

[6] Horiuchi T., Ohkusa T., Watanabe M., Kobayashi D., Miwa H., Eishi Y. (2001). Helicobacter pylori DNA in drinking water in Japan. Microbiol. Immunol..

[7] Fujimura S., Kato S., Watanabe A. (2008). Water source as a Helicobacter pylori transmission route: A 3-year follow-up study of Japanese children living in a unique district. J. Med. Microbiol..

[8] Mazari-Hiriart M., Lopez-Vidal Y., Calva J.J. (2001). Helicobacter pylori in water systems for human use in Mexico City. Water Sci. Technol..

[9] Lu Y.Z., Redlinger T.E., Avitia R., Galindo A., Goodman K. (2002). Isolation and genotyping of Helicobacter pylori from untreated municipal wastewater. Appl. Environ. Microbiol..

[10] Watson C.L., Owen R.J., Said B., Lai S., Lee J.V., Surman-Lee S., Nichols G. (2004). Detection of Helicobacter pylori by PCR but not culture in water and biofilm samples from drinking water distribution systems in England. J. Appl. Microbiol..

[11] Hulten K., Han S.W., Enroth H., Klein P.D., Opekun A.R., Gilman R.H., Evans D.G., Engstrand L., Graham D.Y., ElZaatari F.A.K. (1996). Helicobacter pylori in the drinking water in Peru. Gastroenterology.

[12] Bunn J.E.G., MacKay W.G., Thomas J.E., Reid D.C., Weaver L.T. (2002). Detection of Helicobacter pylori DNA in drinking water biofilms: Implications for transmission in early life. Lett. Appl. Microbiol..

[13] Park S.R., Mackay W.G., Reid D.C. (2001). Helicobacter sp recovered from drinking water biofilm sampled from a water distribution system. Water Res..

[14] Yang F.L., Hassanbhai A.M., Chen H.Y., Huang Z.Y., Lin T.L., Wu S.H., Ho B. (2011). Proteomannans in biofilm of Helicobacter pylori ATCC 43504. Helicobacter.

[15] Ng C.G., Loke M.F., Goh K.L., Vadivelu J., Ho B. (2017). Biofilm formation enhances Helicobacter pylori survivability in vegetables. Food Microbiol..

[16] Leclerc H., Schwartzbrod L., Dei-Cas E. (2002). Microbial agents associated with waterborne diseases. Crit. Rev. Microbiol..

[17] Carter M.J. (2005). Enterically infecting viruses: Pathogenicity, transmission and significance for food and waterborne infection. J. Appl. Microbiol..

[18] Bible J.M., Pantelidis P., Chan P.K.S., Tong C.Y.W. (2007). Genetic evolution of enterovirus 71: Epidemiological and pathological implications. Rev. Med. Virol..

[19] Solomon T., Lewthwaite P., Perera D., Cardoso M.J., McMinn P., Ooi M.H. (2010). Virology, epidemiology, pathogenesis, and control of enterovirus 71. Lancet Infect. Dis..

[20] Wu Y., Yeo A., Phoon M.C., Tan E.L., Poh C.L., Quak S.H., Chow V.T. (2010). The largest outbreak of hand; foot and mouth disease in Singapore in 2008: The role of enterovirus 71 and coxsackievirus A strains. Int. J. Infect. Dis..

[21] Weng K.F., Chen L.L., Huang P.N., Shih S.R. (2010). Neural pathogenesis of enterovirus 71 infection. Microbes Infect..

[22] McMinn P.C. (2002). An overview of the evolution of enterovirus 71 and its clinical and public health significance. FEMS Microbiol. Rev..

[23] Berg G., Sullivan G., Venosa A.D. (1988). Low-temperature stability of viruses in sludges. Appl. Environ. Microbiol..

[24] Marshall B.J., Windsor H.M. (2005). The relation of Helicobacter pylori to gastric adenocarcinoma and lymphoma: Pathophysiology, epidemiology, screening, clinical presentation, treatment, and prevention. Med. Clin. North Am..

[25] Stark R.M., Gerwig G.J., Pitman R.S., Potts L.F., Williams N.A., Greenman J., Weinzweig I.P., Hirst T.R., Millar M.R. (1999). Biofilm formation by Helicobacter pylori. Lett. Appl. Microbiol..

[26] García A., Salas-Jara M.J., Herrera C., González C. (2014). Biofilm and Helicobacter pylori: From environment to human host. World J. Gastroenterol..

[27] Hathroubi S., Servetas S.L., Windham I., Merrell D.S., Ottemann K.M. (2018). Helicobacter pylori biofilm formation and its potential role in pathogenesis. Microbiol. Mol. Biol. Rev..

[28] Krzyzek P., Migdal P., Grande R., Gosciniak G. (2022). Biofilm formation of Helicobacter pylori in both static and microfluidic conditions is associated with resistance to clarithromycin. Front. Cell Infect. Microbiol..

[29] Al-Maleki A.R., Loke M.F., Lui S.Y., Ramli N.S.K., Khosravi Y., Ng C.G., Venkatraman G., Goh K.L., Ho B., Vadivelu J. (2017). Helicobacter pylori outer inflammatory protein A (OipA) suppresses apoptosis of AGS gastric cells in vitro. Cell Microbiol..

[30] Quignon F., Sardin M., Kiene L., Schwartzbrod L. (1997). Poliovirus-1 inactivation and interaction with biofilm: A pilot-scale study. Appl. Environ. Microbiol..

[31] Storey M.V., Ashbolt N.J. (2001). Persistence of two model enteric viruses (B40-8 and MS-2 bacteriophages) in water distribution pipe biofilms. Water Sci. Technol..

[32] Storey M.V., Ashbolt N.J. (2003). Enteric virions and microbial biofilms–A secondary source of public health concern?. Water Sci. Technol..

[33] Tan C.W., Poh C.L., Sam I.C., Chan Y.F. (2013). Enterovirus 71 uses cell surface heparan sulfate glycosaminoglycan as an attachment receptor. J. Virol..

[34] Lin Y.C., Lin S.T., Chen C.Y., Wu S.C. (2012). Enterovirus 71 adsorption on metal ion-composite chitosan beads. Biotechnol. Prog..

[35] Myint S., Manley R., Cubitt D. (1994). Viruses in bathing waters. Lancet.

[36] Mackay W.G., Gribbon L.T., Barer M.R., Reid D.C. (1999). Biofilms in drinking water systems: A possible reservoir for Helicobacter pylori. J. Appl. Microbiol..

[37] Ali M.A., Al-Herrawy A.Z., El-Hawaary S.E. (2004). Detection of enteric viruses, Giardia and Cryptosporidium in two different types of drinking water treatment facilities. Water Res..

[38] Vivier J.C., Ehlers M.M., Grabow W.O. (2004). Detection of enteroviruses in treated drinking water. Water Res..

[39] Ehlers M.M., Grabow W.O., Pavlov D.N. (2005). Detection of enteroviruses in untreated and treated drinking water supplies in South Africa. Water Res..

[40] Ranganathan S., Singh S., Poh C.L., Chow V.T. (2002). The hand, foot and mouth disease virus capsid: Sequence analysis and prediction of antigenic sites from homology modelling. Appl. Bioinform..

[41] Lal S.K., Kumar P., Yeo W.M., Kar-Roy A., Chow V.T. (2006). The VP1 protein of human enterovirus 71 self-associates via an interaction domain spanning amino acids 66-297. J. Med. Virol..

[42] Sinclair R.G., Jones E.L., Gerba C.P. (2009). Viruses in recreational water-borne disease outbreaks: A review. J. Appl. Microbiol..

[43] Love D.C., Rodriguez R.A., Gibbons C.D., Griffith J.F., Yu Q.L., Stewart J.R., Sobsey M.D. (2014). Human viruses and viral indicators in marine water at two recreational beaches in Southern California, USA. J. Water Health.

[44] Cabelli V.J., Dufour A.P., Mccabe L.J., Levin M.A. (1982). Swimming-associated gastroenteritis and water quality. Am. J. Epidemiol..

[45] Seyfried P.L., Tobin R.S., Brown N.E., Ness P.F. (1985). A prospective study of swimming-related illness. I. Swimming-associated health risk. Am. J. Public Health.

[46] Craun G.F., Calderon R.L., Craun M.F. (2005). Outbreaks associated with recreational water in the United States. Int J. Environ. Health Res..

[47] Cheung W.H., Chang K.C., Hung R.P., Kleevens J.W. (1990). Health effects of beach water pollution in Hong Kong. Epidemiol. Infect..

[48] Hughes M.S., Coyle P.V., Connolly J.H. (1992). Enteroviruses in recreational waters of Northern Ireland. Epidemiol. Infect..

[49] Corbett S.J., Rubin G.L., Curry G.K., Kleinbaum D.G. (1993). The health effects of swimming at Sydney beaches. The Sydney Beach Users Study Advisory Group. Am. J. Public Health.

[50] Lee C.S., Lee C., Marion J., Wang Q., Saif L., Lee J. (2014). Occurrence of human enteric viruses at freshwater beaches during swimming season and its link to water inflow. Sci. Total Environ..

[51] Skraber S., Schijven J., Gantzer C., de Roda Husman A.M. (2005). Pathogenic viruses in drinking-water biofilms: A public health risk?. Biofilms.

[52] Lodder W.J., Vinje J., van De Heide R., de Roda Husman A.M., Leenen E.J., Koopmans M.P. (1999). Molecular detection of Norwalk-like caliciviruses in sewage. Appl. Environ. Microbiol..

[53] Skraber S., Ogorzaly L., Helmi K., Maul A., Hoffmann L., Cauchie H.M., Gantzer C. (2009). Occurrence and persistence of enteroviruses, noroviruses and F-specific RNA phages in natural wastewater biofilms. Water Res..

[54] Smith E.M., Gerba C.P., Melnick J.L. (1978). Role of sediment in the persistence of enteroviruses in the estuarine environment. Appl. Environ. Microbiol..

[55] Sakoda A., Sakai Y., Hayakawa K., Suzuki M. (1997). Adsorption of viruses in water environment onto solid surfaces. Water Sci. Technol..

[56] Karim M.R., Manshadi F.D., Karpiscak M.M., Gerba C.P. (2004). The persistence and removal of enteric pathogens in constructed wetlands. Water Res..

[57] Helmi K., Skraber S., Gantzer C., Willame R., Hoffmann L., Cauchie H.M. (2008). Interactions of Cryptosporidium parvum, Giardia lamblia, vaccinal poliovirus type 1, and bacteriophages phiX174 and MS2 with a drinking water biofilm and a wastewater biofilm. Appl. Environ. Microbiol..

[58] O’Toole G.A., Kolter R. (1998). Initiation of biofilm formation in Pseudomonas fluorescens WCS365 proceeds via multiple, convergent signalling pathways: A genetic analysis. Mol. Microbiol..

[59] Singh S., Chow V.T., Phoon M.C., Chan K.P., Poh C.L. (2002). Direct detection of enterovirus 71 (EV71) in clinical specimens from a hand, foot, and mouth disease outbreak in Singapore by reverse transcription-PCR with universal enterovirus and EV71-specific primers. J. Clin. Microbiol..

[60] Singh S., Poh C.L., Chow V.T. (2002). Complete sequence analyses of enterovirus 71 strains from fatal and non-fatal cases of the hand, foot and mouth disease outbreak in Singapore (2000). Microbiol. Immunol..

[61] Yeo H., Chong C.W.H., Chen E.W., Lim Z.Q., Ng Q.Y., Yan B., Chu J.J.H., Chow V.T.K., Alonso S. (2022). A single amino acid substitution in structural protein VP2 abrogates the neurotropism of enterovirus A-71 in mice. Front. Microbiol..

[62] Hassanbhai A.M. (2012). Characterization of Biofilm Formation by Helicobacter Pylori. PhD Thesis.

