# Supplementary material for: The Association of Helicobacter pylori Biofilm with Enterovirus 71 Prolongs Viral Viability and Survival
Source: Int J Mol Sci. 2023 Sep 24;24(19):14500. doi: 10.3390/ijms241914500 (PMC10572889; doi:10.3390/ijms241914500)
Supplement: Supplementary file 1 [file ijms-24-14500-s001.zip › ijms-2449127-supplementary.pdf]

A

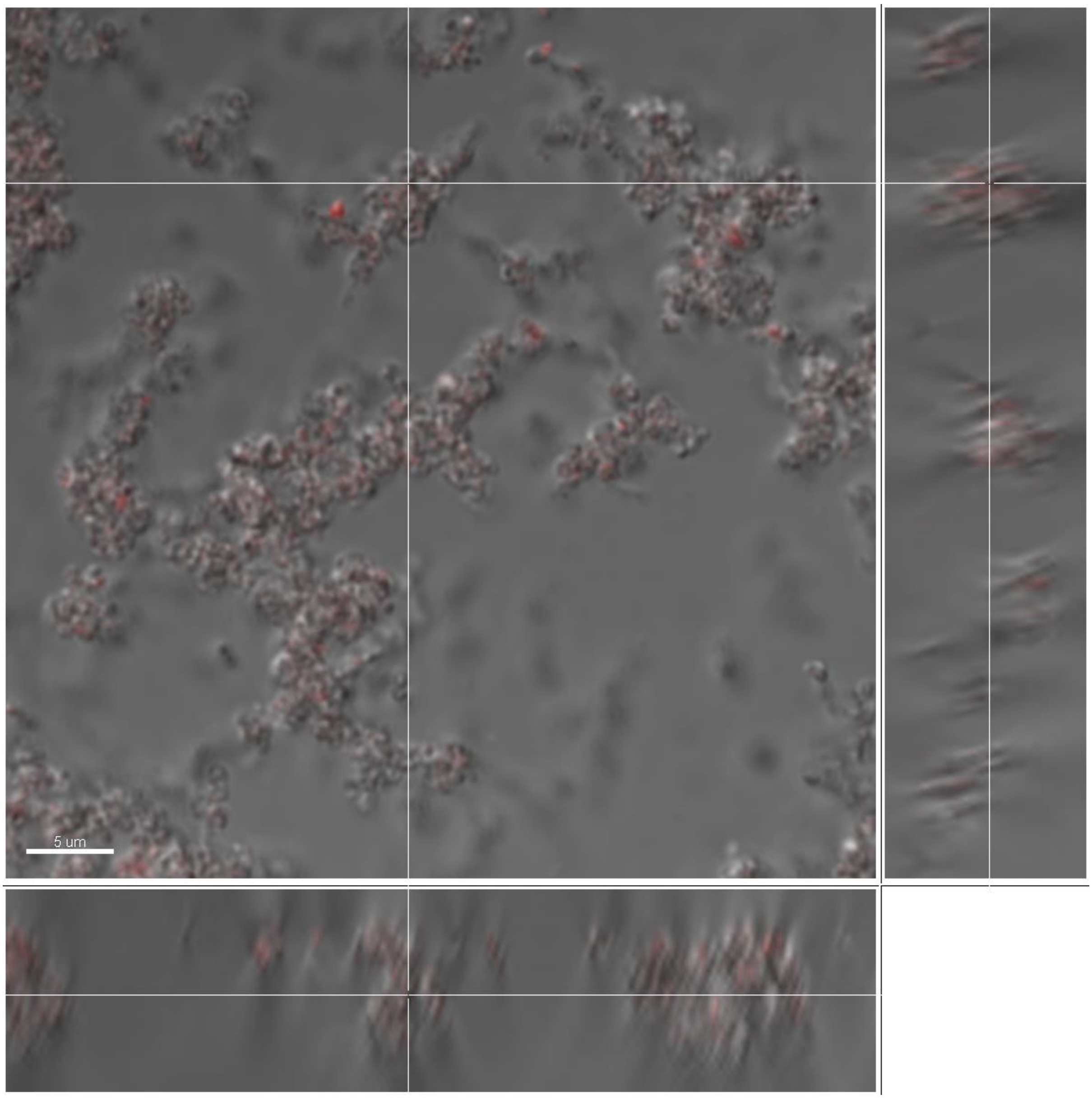

B

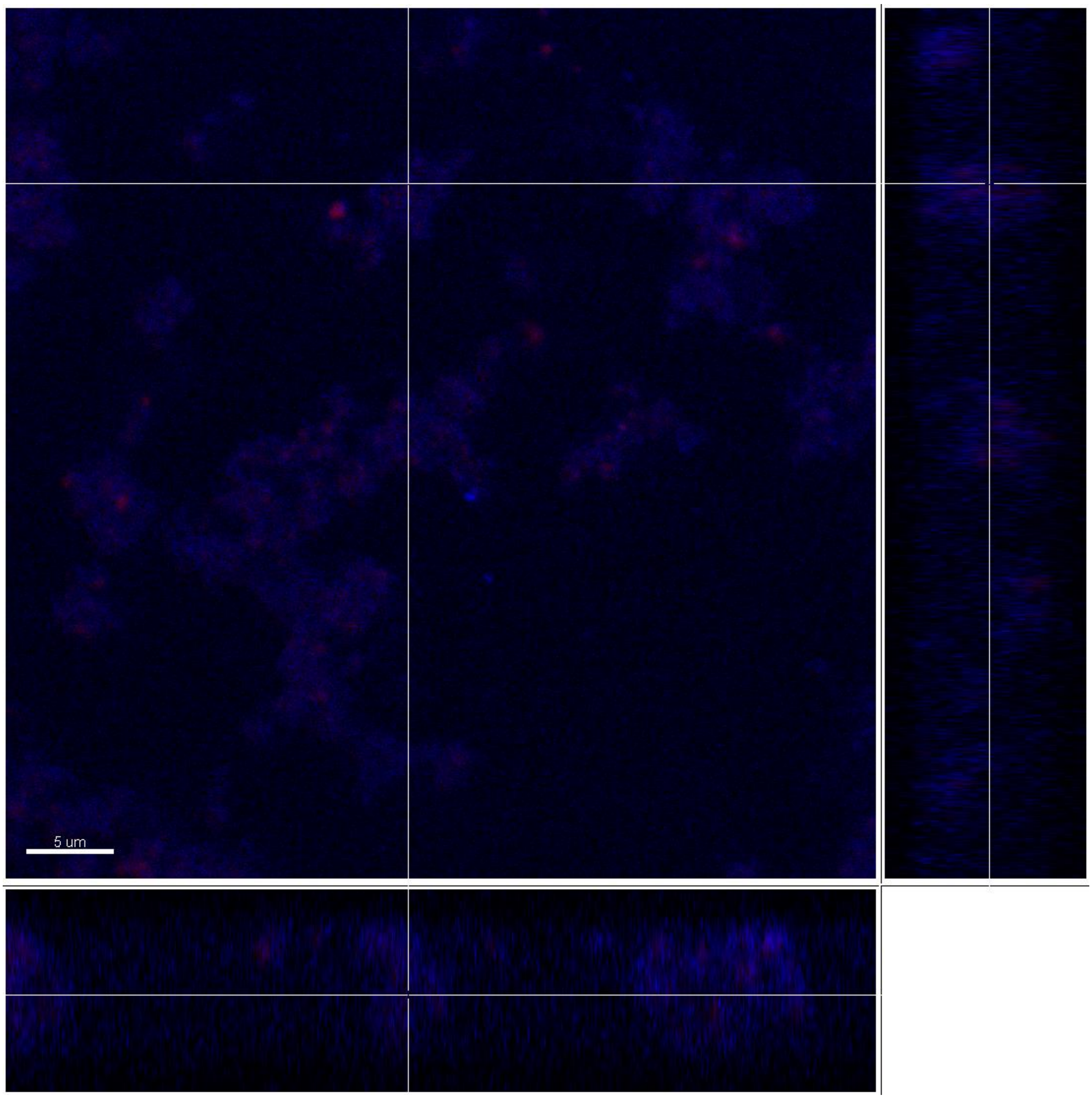

C

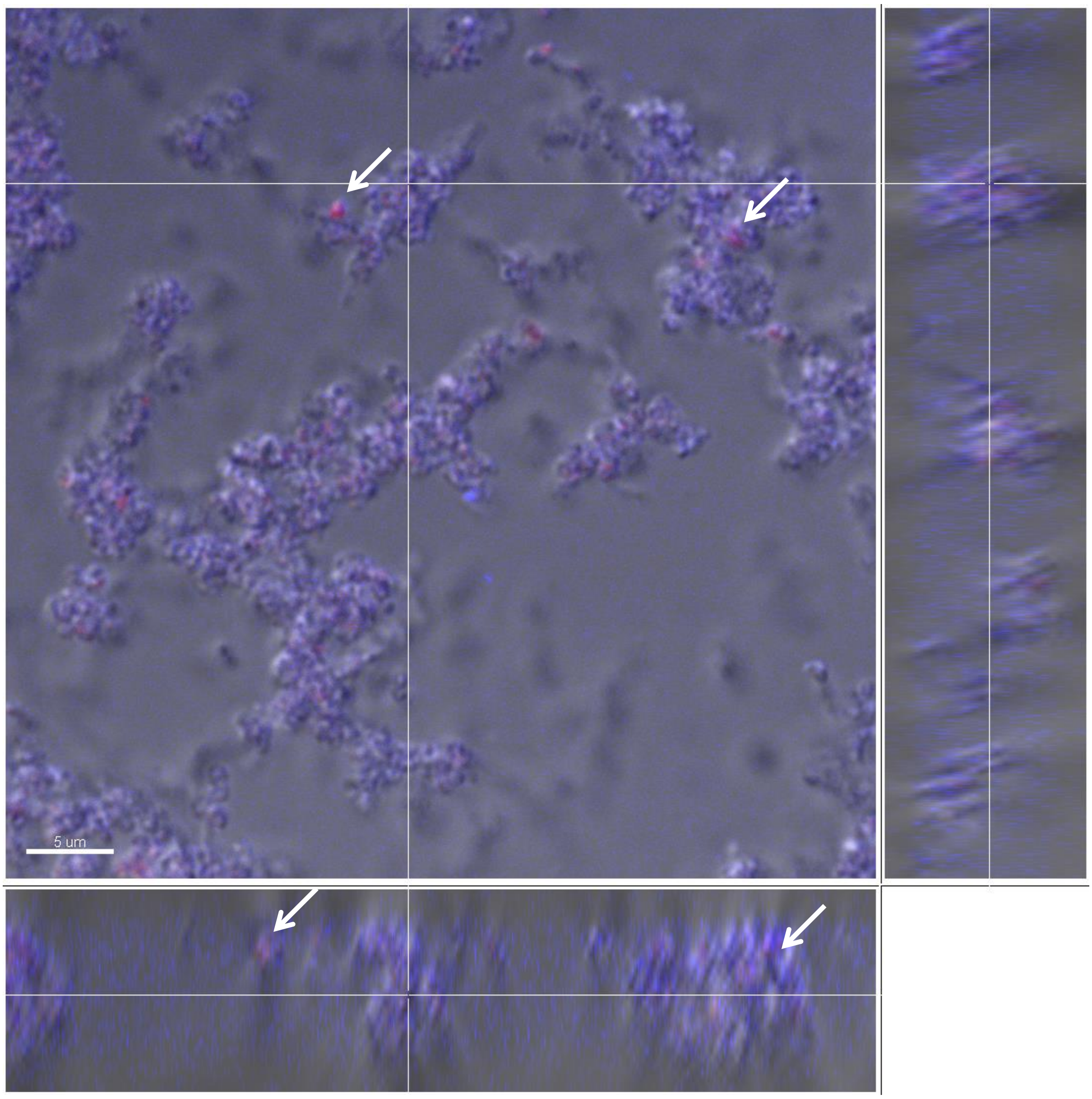

Supplementary Figure S1. Cross-sectional confocal microscopy view of co-incubation of EV71 with one week-old biofilm of *H. pylori* NCTC11637 strain after 3 days. (A) Brightfield image with the EV71 particles appearing red from immunolabeling with an EV71-specific primary antibody followed by a secondary antibody conjugated to Cy3. (B) Image depicting blue staining of *H. pylori* biofilm by Calcofluor. (C) Merged images of (A) and (B). This *H. pylori* strain generated prominent three-dimensional, thick and dense biofilm structures, with multi-layered microcolonies. The virus particles (in red as shown by representative white arrows) can be observed as randomly distributed across the biofilm (stained blue). Horizontal and vertical thin white lines represent the x axis and y axis across sectional images, respectively. The images were rendered using Imaris. Scale bar represents 5 μm.
